# Editorial: Allosteric functions and inhibitions: structural insights

**DOI:** 10.3389/fmolb.2024.1363100

**Published:** 2024-01-15

**Authors:** David D. Boehr

**Affiliations:** Department of Chemistry, The Pennsylvania State University, University Park, PA, United States

**Keywords:** allostery, conformational dynamics, conformational ensemble, network model, protein-ligand binding

The fundamental principle of allostery, known as “action from a distance,” is crucial in biological regulation, as illustrated by historical examples in gene regulation and enzymatic pathways. Traditional allostery models like the concerted Monod-Wyman-Changeux (MWC) (Monod et al., 1965) and sequential Koshland-Nemethy-Filmer (KNF) (Koshland et al., 1966) have evolved into more comprehensive frameworks, incorporating insights from protein conformational ensembles (Motlagh et al., 2014). The definition of allostery has expanded to include remote structural and/or dynamic changes induced by ligands, including small molecules and macromolecular binding partners, covalent modifications, and amino acid substitutions. This Research Topic aimed to spotlight emerging trends in allosteric regulation, particularly focusing on advancements in structure- and biophysics-driven methods.

Hur et al. Original Research article sheds light on resveratrol’s diverse effects on SIRT1, a NAD^+^-dependent deacetylase. SIRT1 allosteric regulation has been investigated as a potential therapeutic approach, due to its role in various diseases, including Alzheimer’s and Type II diabetes (Sinclair and Guarente, 2014; Manjula et al., 2021). Examination of over 6,800 acetylated peptide substrates revealed resveratrol’s varied impact—acting as an activator, inhibitor, or having no effect, depending on the substrate (Lakshminarasimhan et al., 2013). In the present work, enzyme kinetic studies revealed resveratrol’s modulation of the Michaelis-Menten parameter (K_M_) without altering the catalytic rate (k_cat_), indicating changes in substrate recognition and affinity. Additionally, resveratrol induced protein instability and altered conformations, with outcomes dependent on the peptide substrate. The authors propose a model elucidating resveratrol’s dual role: as an activator, it enhances SIRT1 flexibility, improving efficiency; as an inhibitor, it unravels crucial structural elements, impairing substrate recognition.

The Original Research article by (Venkatakrishnan et al.) investigated acrodysostosis-linked mutations in the protein kinase A (PKA) type 1 regulatory subunit isoform alpha (R1α) and associated phosphodiesterase (PDE). Acrodysostosis comprises rare genetic disorders with diverse clinical features, including skeletal anomalies and cognitive impairments (Silve et al., 2012). PKA activation is initiated by cAMP binding to the R subunit and PDE-mediated cAMP hydrolysis then acts to return PKA to its original inhibited state (Jarnaess and Taskén, 2007). The mutations, T207A in R1α and T690P in PDE8, are located at the R1α:PDE8 interface. Both mutations impaired processive cAMP cycling. Based on hydrogen-deuterium exchange mass spectrometry (HDXMS) analysis, the authors suggest that the T690P mutation diminishes or eliminates the conformational dynamics linked to the nucleotide channel in PDE8, which may act as both an ingress and egress point for cAMP and AMP respectively. The authors also propose that the interactions between the side chain of Thr207 and the phosphate moiety of cAMP or AMP play a crucial role in the efficient channeling of cAMP out of the system; the loss of these interactions likely slows the channeling process.

The Original Research article by (Lee et al.) investigated structural and conformational dynamic changes in the enzyme biliverdin reductase B (BLVRB) throughout its catalytic cycle. BLVRB, a NADPH-dependent oxidoreductase, plays a critical role in redox regulation and is implicated in hepatic and prostate cancer (Huan et al., 2016; Ramberg et al., 2021). X-ray crystallography and nuclear magnetic resonance (NMR) studies revealed structural and dynamic changes across catalytic intermediates. Residues displaying chemical shift differences between the enzyme bound with the oxidized and reduced cofactor were mutated, including those near (e.g., Ser111 and His153) and far (i.e., Thr164) from the active site. These mutations all induced changes in enzyme kinetics, prompting the authors to propose that allosteric coupling to the cofactor’s oxidation state is crucial for enzyme function. R_2_ relaxation dispersion experiments, which measure conformational dynamics on the μs-ms timescale (Farber and Mittermaier, 2015), identified distinct residue dynamics, with one group exhibiting motion only in the apo state and another throughout the rest of the catalytic cycle. A third group, including Thr164, displayed differences between oxidized and reduced cofactor states, and may be important in relaying the allosteric effect of the T162S mutation.

The Review article by (Knight et al.) highlights the insights gained from comparing mesophilic and thermophilic enzyme counterparts, shedding light on conformational ensembles and intra-protein communication. The authors discuss findings from temperature-dependent NMR, X-ray crystallography, and calorimetry studies, revealing that temperature changes can enhance, suppress, or have no effect on allosteric coupling. So, while studies have brought insight into individual cases, the authors note that there is a lack of broadly defined biophysical principles that distinguish mesophilic from thermophilic proteins, which would help propel the field further.

The Review article by (Komives) focuses on the enzyme thrombin, a serine protease that plays a key regulatory role in the blood coagulation pathway (Di Cera et al., 2007). Interestingly, binding of Na^+^ favors the cleavage of fibrinogen to form the fibrin clot (procoagulant activity), whereas thrombomodulin (TM) binding favors the proteolytic activation of protein kinase C (anticoagulant activity). The review highlights what has been learned from HDXMS, NMR and computational studies of thrombin, especially revealing functionally-relevant conformational dynamics across ns-s timescales. The TM binding site of thrombin is shown to be allosterically coupled to the active site, where TM binding increases and decreases protein motions in the N- and C-terminal β-barrels, respectively, to modulate function.

The Review article by (Yu and Boehr) summarizes recent findings on how membrane interactions impact the structure, dynamics, and function of peripheral-membrane binding enzymes. These studies utilize various methods including X-ray crystallography, NMR, HDXMS, and molecular dynamics simulations. Despite significant insights into the allosteric activation of these enzymes, more detailed understanding of structural rearrangements awaits higher resolution structure determination in most cases. In-cell imaging methods can complement these studies, offering insights into the colocalization of membrane-bound proteins with specific lipids, leading to allosteric regulation.

These articles emphasize diverse structural and biophysical methods employed to investigate protein allostery, which integrate well with computational models. They highlight the dual importance of both structural alterations and changes in internal protein motions to govern enzyme function. Exploring these enzymes further will aid in identifying and utilizing allosteric sites, conventional and cryptic, which offer novel drug interaction sites for therapeutic interventions. Moreover, similar studies in relevant cases can establish a groundwork for protein engineering in applications like biotechnology, synthetic biology, and medicine.
